# Multimodal Approach for the Prediction of Atrial Fibrillation Detected After Stroke: SAFAS Study

**DOI:** 10.3389/fcvm.2022.949213

**Published:** 2022-07-13

**Authors:** Lucie Garnier, Gauthier Duloquin, Alexandre Meloux, Karim Benali, Audrey Sagnard, Mathilde Graber, Geoffrey Dogon, Romain Didier, Thibaut Pommier, Catherine Vergely, Yannick Béjot, Charles Guenancia

**Affiliations:** ^1^Department of Neurology, University Hospital, Dijon, France; ^2^Pathophysiology and Epidemiology of Cerebro-Cardiovascular Diseases (EA 7460), Faculty of Health Sciences, Université de Bourgogne, Université de Bourgogne Franche-Comté, Dijon, France; ^3^Department of Cardiology, University Hospital, Dijon, France

**Keywords:** atrial fibrillation, stroke, atrial cardiopathy, biomarkers, Holter, echocardiography

## Abstract

**Background:**

Intensive screening for atrial fibrillation (AF) has led to a better recognition of this cause in stroke patients. However, it is currently debated whether AF Detected After Stroke (AFDAS) has the same pathophysiology and embolic risk as prior-to-stroke AF. We thus aimed to systematically approach AFDAS using a multimodal approach combining clinical, imaging, biological and electrocardiographic markers.

**Methods:**

Patients without previously known AF admitted to the Dijon University Hospital (France) stroke unit for acute ischemic stroke were prospectively enrolled. The primary endpoint was the presence of AFDAS at 6 months, diagnosed through admission ECG, continuous electrocardiographic monitoring, long-term external Holter during the hospital stay, or implantable cardiac monitor if clinically indicated after discharge.

**Results:**

Of the 240 included patients, 77 (32%) developed AFDAS. Compared with sinus rhythm patients, those developing AFDAS were older, more often women and less often active smokers. AFDAS patients had higher blood levels of NT-proBNP, osteoprotegerin, galectin-3, GDF-15 and ST2, as well as increased left atrial indexed volume and lower left ventricular ejection fraction. After multivariable analysis, galectin-3 ≧ 9 ng/ml [OR 3.10; 95% CI (1.03–9.254), *p* = 0.042], NT-proBNP ≧ 290 pg/ml [OR 3.950; 95% CI (1.754–8.892, *p* = 0.001], OPG ≥ 887 pg/ml [OR 2.338; 95% CI (1.015–5.620), *p* = 0.046) and LAVI ≥ 33.5 ml/m^2^ [OR 2.982; 95% CI (1.342–6.625), *p* = 0.007] were independently associated with AFDAS.

**Conclusion:**

A multimodal approach combining imaging, electrocardiography and original biological markers resulted in good predictive models for AFDAS. These results also suggest that AFDAS is probably related to an underlying atrial cardiopathy.

**Clinical Trial Registration:**

[www.ClinicalTrials.gov], identifier [NCT03570060].

## Introduction

Atrial fibrillation (AF) is one of the most common causes of ischemic stroke. It is associated with a fivefold increased risk of stroke and accounts for more than one in five strokes (1, 2). However, many studies showed that even with continuous electrocardiographic monitoring (CEM), many AF episodes are not clinically diagnosed (3). It has been estimated that one third of patients with cryptogenic stroke may in fact have undetected asymptomatic AF (1, 4). In this setting, implantable cardiac monitors (ICM) are now commonly implanted in patients with cryptogenic stroke, in accordance with CRYSTAL-AF trial results (1). However, only one third of patients with ICM will eventually develop new-onset AF (5). Systematic anticoagulation in cryptogenic stroke patients failed to demonstrate a clinical benefit as compared to aspirin in the NAVIGATE-ESUS or RESPECT-ESUS trials (6, 7), supporting the emergence of a new concept known as AFDAS (Atrial Fibrillation Detected After Stroke and Transient Ischemic Attack). Although some studies tend to show that AFDAS has a different pathophysiology and embolic risk than AF, little is known about the underlying mechanisms of this condition (8).

Three components are involved in the development of all types of arrhythmia (9): the substrate, the modulator [the autonomic nervous system (ANS)] and the triggering factor(s). We thus aimed to approach systematically AFDAS pathophysiology in stroke patients using a multimodal approach combining clinical, imaging, biological and electrocardiographic markers:

- the atrial substrate (i.e. atrial cardiopathy), as assessed by left atrial dimensions, and by blood biomarkers of fibrosis (galectin-3, osteoprotegerin) and of cardiac remodeling (NT-pro-BNP),
- the modulator: ANS, assessed by heart rate variability,
- the triggers, as assessed by inflammatory mediators (CRP, ST2, GDF-15), the burden of premature atrial contractions and the presence of left ventricular dysfunction or acute myocardial injury.

### Study Design and Population

We conducted a prospective study (SAFAS: Stepwise screening for silent Atrial Fibrillation After Stroke) in adult patients hospitalized between March 31, 2018, and January 18, 2020, in the stroke unit of the Dijon Bourgogne University Hospital. We included patients with ischemic stroke according to the World Health Organization criteria: a clinical syndrome characterized by a focal loss of cerebral or ocular function, of sudden onset, without obvious etiology at initial management and confirmed by imaging.

Patients with a history of AF or atrial flutter, as well as those with a pacemaker or an implantable cardioverter defibrillator with an atrial lead (not eligible for the stepwise screening strategy), adults under guardianship, pregnant or breastfeeding women, and those who refused to participate in the study were excluded. Patients who were not under the primary care area of the Dijon University Hospital (transfer to a department outside the University Hospital after acute management of stroke) were excluded because long-term external Holter screening could not be done.

Oral consent was obtained from all patients or their representative. This study was validated by a national ethics committee and conducted in accordance with the ethical principles of the Declaration of Helsinki and the recommendations of Good Clinical Practice (CPP Sud Méditerranée I n°2018-A00345-50, clinical trials NCT03570060).

### Clinical, Biological and Imaging Data During Hospital Stay

Within 48 h of admission, we collected patients’ demographic and clinical data. Upon admission to the stroke unit, patients underwent additional examinations, including brain and intra/extra-cranial vessel imaging, electrocardiogram (ECG), echocardiography [transthoracic with bubble test for patent foramen ovale (PFO) ± transesophageal] and a standard biological workup supplemented by sampling for biomarkers.

### Biomarker Assay

Biological samples were stored in the stroke unit refrigerator at 3°C for a maximum of 24 h. Tubes were centrifuged at 3,500 rpm for 5 min at 4°C to recover the serum, and then aliquoted. The aliquots were then immediately transferred to the freezer (−80°C) until use. The assay was performed on thawed serum. The Enzyme-Linked Immuno-Sorbent Assay technique was used for galectin-3 (DGAL30, R&D systems, Minneapolis, United States) and the multiplex technique for osteoprotegerin (OPG), ST2, and GDF15 (R&D systems, Minneapolis, United States) following the manufacturer’s recommendations.

### Heart Rate Monitoring

Patients received continuous and sequential cardiac monitoring which included ECG at admission, CEM in the stroke unit, and long-term Holter ECG (SpiderFlash, Microport, France) during the entire stay in the neurology department. The SpiderFlash Holter allows the recording of arrhythmic episodes regardless of their duration, capturing the events and documenting them by means of ECG samples. The device was programmed to record every rhythmic event for 7 days, regardless of the duration of the supraventricular arrhythmia. An experienced cardiologist (CG) who was blinded to the patient’s clinical data performed the Holter ECG analysis. If the diagnosis was uncertain, a second cardiologist, blinded to the first results, also analyzed the records. There was no discordance between the two analyses.

If no arrhythmia was detected and no etiology found for the ischemic stroke after the diagnostic workup, an ICM (REVEAL XT or Linq, Medtronic, United States) was indicated, as recommended by international guidelines in case of cryptogenic stroke (10). ICM detects AF by analyzing the irregularity and inconsistency of successive R-R intervals within a minimum time frame (5). Atrial fibrillation was defined according to European guidelines (11). Atrial flutter patients were included in the AF group.

### Electrocardiographic Data

*ECG: P*-wave duration (ms) and *p*-wave terminal force (PTF) [amplitude of the terminal negative portion of the P-wave in V1 x the duration of the terminal negative portion of the *P*-wave in V1)].

*Heart rate variability (HRV) on CEM tracings* were measured as previously described (12): average pNN50 (marker of parasympathetic nervous system activation) corresponding to the proportion derived by dividing NN50 [the number of interval differences of successive sinus node depolarization (NN) intervals greater than 50 ms] by the total number of NN intervals, and SDNN [the standard deviation of all intervals between adjacent QRS complexes resulting from sinus node depolarization (NN)], on the first day of recording of the stroke unit CEM. Only sequences with normal QRS characteristics during 24 h (sinus rhythm) were analyzed for HRV study. If AF was diagnosed on the ECG at entry or on the first day of monitoring, ANS parameters were not analyzed.

### Follow-Up

We collected the length of stay in each unit, where the patient was discharged to after hospitalization, any intercurrent hospital events, etiological diagnosis according to the TOAST classification (13) as well as the NIHSS score, modified Rankin score and discharge treatments.

Patients were contacted by phone at 3 months and seen for an outpatient visit at 6 months. Data was collected regarding vital status, current treatments, cardiovascular events (ischemic stroke, myocardial infarction, heart failure hospitalization, atrial fibrillation or atrial flutter), vascular or hemorrhagic recurrence, and any hemorrhagic event.

Patients implanted with an ICM had a follow-up cardiology consultation at 6 weeks and then every 3 months. If the patient was equipped with a remote monitoring system, the ICM data were analyzed every week and the patient was contacted if a rhythm disorder was detected. Decisions regarding the treatment of AF episodes were made by the attending physician.

### Statistical Analysis

Continuous data were expressed as medians (25th–75th percentile) and dichotomous data as numbers (percentages). A Mann-Whitney test or Student’s *t*-test was used to compare continuous data, and the Chi-square test or Fisher’s test was used for dichotomous data. The optimal threshold to discriminate AF from the continuous data of interest was obtained with the receiver-operating characteristic (ROC) curve with the best sensitivity and specificity according to the Youden index. Variables entered into the multivariate model were chosen according to their univariate relationship with an inclusion and exclusion cut-off at 5%. Two multivariate backward stepwise logistic regression models were used, one to predict all recorded AFDAS from admission to 6-month follow-up (model 1) and the second one focusing on AFDAS diagnosed after the stay in the stroke unit, including HRV variables (model 2). A *p*-value < 0.05 was considered statistically significant. Analyses were performed using SPSS software (26.0, IBM Inc., United States).

## Results

### Atrial Fibrillation Detected After Stroke Associated Factors

**FIGURE 1:**
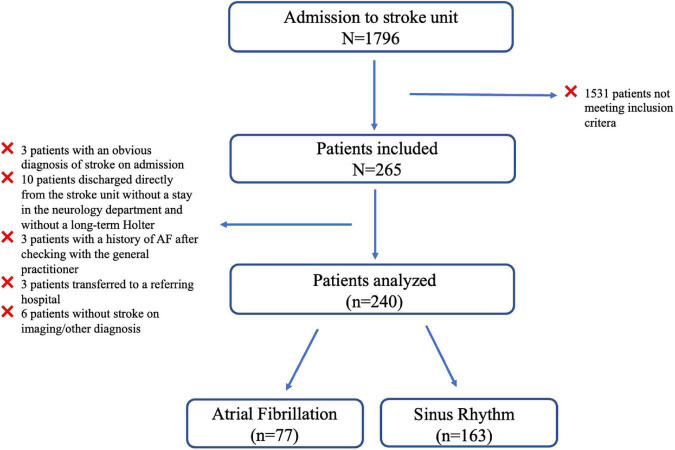
Flow chart of the SAFAS study.

Among the 1,796 patients admitted to the stroke unit between March 2018 and January 2020, 265 were eligible for inclusion, and 240 were finally analyzed (Figure 1). During the 6 months of follow-up, 77 patients (32%) developed AFDAS and 163 patients (64%) maintained sinus rhythm. Clinical characteristics and complementary exam results are presented in Tables 1–4.

**TABLE 1:** Clinical characteristics of patients [*n* (%) or median (IQR)].

	SR [*n* = 163 (68%)]	AFDAS [*n* = 77 (32%)]	*p*
**Risk factors**			
Age, years	65.79 (54.90–74.96)	81.27 (71.68–85.85)	< 0.001
Age ≥ 77 years	31.00 (19.00)	49.00 (63.30)	< 0.001
Female sex	68.00 (41.70)	45.00 (58.40)	0.015
BMI, kg/m^2^	26.26 (23.75–29.19)	26.44 (23.10–29.79)	0.778
Obesity (BMI > 30 kg/m^2^)	35.00 (21.90)	16.00 (22.9)	0.869
High blood pressure	86.00 (52.80)	50.00 (64.90)	0.076
Hypercholesterolemia	49.00 (30.10)	21.00 (27.30)	0.657
Diabetes	30.00 (18.40)	16.00 (20.80)	0.663
Active smoking	41.00 (25.20)	6.00 (7.90)	0.001
Active or withdrawn alcohol consumption	13.00 (8.00)	5.00 (6.70)	0.799
Obstructive sleep apnea	14.00 (8.60)	9.00 (11.80)	0.483
Previous kidney failure	2.00 (1.20)	3.00 (3.90)	0.331
Previous cancer	26.00 (16.00)	10.00 (13.00)	0.548
Recent infection (<1 month)	6.00 (3.70)	6.00 (7.90)	0.206
**Cardiovascular history**			
Stroke or TIA	25.00 (15.50)	13.00 (17.10)	0.757
Peripheral artery disease	2,00 (1.20)	1,00 (1.30)	1,000
Heart failure	5.00 (3.10)	5.00 (6.60)	0.296
Cardiac valve disease	7,00 (4.30)	6.00 (7.90)	0.357
**Clinical data at admission**			
Systolic pressure, mmHg	155.00 (138.25–175.00)	161.00 (139.50–178.50)	0.910
Diastolic pressure, mmHg	87.00 (75.25–95.00)	81.00 (70.00–92.50)	0.061
Blood glucose, g/l	1.17 (1.02–1.37)	1.11 (0.99–1.35)	0.288
NIHSS score	4.00 (1.00–7.00)	6.00 (3.00–12.75)	0.001
Premorbid mRS ≥ 2	20.00 (12.70)	28.00 (37.30)	< 0.001
CHA_2_DS_2_VASc score	2.00 (1.00–4.00)	4.00 (2.00–4.00)	< 0.001
Acute revascularization therapy	54.00 (33.10)	42.00 (54.50)	0.002

*IQR, interquartile range; SR, sinus rhythm; BMI, Body mass index; TIA, transient ischemic attack; NIHSS, National Institute of Health Stroke Scale; mRS, modified Rankin scale.*

**TABLE 2:** Biological, imaging and electrocardiographic characteristics of patients at admission [*n* (%) or median (IQR)].

	Sinus rhythm	AFDAS	*p*
**Biological data**			
CRP, mg/mL	2.90 (2.90–5.00)	2.95 (2.90–6.10)	0.269
Creatinine, μmol/l	72.00 (62.00–85.00)	74.00 (63.00–88.00)	0.476
Troponin, μg/l	0.02 (0.02–0.02)	0.02 (0.02–0.04)	0.417
NT-Pro-BNP, pg/ml	129.00 (60.00–331.00)	843.00 (303.25–2069.50)	< 0.001
NT-pro-BNP ≥ 290 pg/ml	43.00 (28.70)	54.00 (77.10)	< 0.001
TSH, μg/ml	1.32 (0.76–2.07)	1.16 (0.78–1.88)	0.772
Galectin 3, ng/ml	11.03 (8.24–14.33)	12.45 (10.03–16.29)	0.026
Galectin 3 ≥ 9 ng/ml	106.00 (68.40)	68.00 (88.30)	0.001
ST2, pg/ml	17147.90 (12932.97–26956.95)	21.202.68 (14708.56–31836.67)	0.027
ST2 ≥ 18,350 pg/ml	63.00 (39.90)	48.00 (63.20)	0.001
Osteoprotegerin, pg/ml	905.60 (757.07–1280.77)	1139.30 (912.92–1598.76)	< 0.001
Osteoprotegerin ≥ 887 pg/ml	80.00 (50.60)	60.00 (77.90)	< 0.001
GDF15, pg/ml	1573.64 (1003.80–2270.86)	2142.63 (1363.14-2931.12)	0.001
GDF15 ≥ 1,320 pg/ml	94.00 (59.90)	61.00 (79.20)	0.003
**Imaging data**			
Multi-territory stroke	19.00 (11.70)	6.00 (7.80)	0.360
Vertebro-basilar stroke	58.00 (35.60)	18.00 (23.40)	0.058
Bilateral stroke	15.00 (9.20)	6.00 (7.80)	0.718
Insular stroke	27.00 (16.60)	26.00 (33.80)	0.003
Cerebellar stroke	18.00 (11.00)	7.00 (9.10)	0.821
Thalamic stroke	9.00 (5.50)	5.00 (6.50)	0.773
Anterior choroidal stroke	10.00 (6.10)	0.00 (0.00)	0.033
CAA stroke	6.00 (3.70)	4.00 (5.20)	0.730
MCA superficial stroke	73.00 (44.80)	53.00 (68.80)	< 0.001
MCA deep stroke	43.00 (26.40)	22.00 (28.60)	0.721
**ECG data**			
LBB (*n* = 180)	3.00 (2.40)	2.00 (3.50)	0.685
RBB (*n* = 180)	7.00 (5.70)	5.00 (8.80)	0.523
*P*-wave duration max, ms (*n* = 111)	100.00 (100.00–120.00)	120.00 (100.00–120.00)	0.570
PTF, mV.ms	4.00 (4.00–8.00)	6.00 (4.00–8.00)	0.407
PTF ≥ 4 mv.ms (*n* = 111)	41.00 (47.10)	17.00 (70.80)	0.063
PTF ≥ 5 mv.ms (*n* = 111)	38.00 (46.30)	20.00 (69.00)	0.051
PR duration, ms	168.00 (148.00–191.00)	172.00 (154.00–196.00)	0.214
QRS duration, ms	90,00 (82.50–98.00)	92.00 (82.00–108.00)	0.385
Corrected QTc duration, ms	423.00 (409.00–444.00)	435.00 (413.50–449.50)	0.189

*IQR, interquartile range; SR, sinus rhythm; AF, Atrial fibrillation CRP, C-reactive protein; NT-pro-BNP, N-Terminal pro-brain natriuretic peptide; ST2, Suppression of Tumorigenicity 2; GDF15, growth differentiation factor 15; cerebral anterior artery; MCA, middle cerebral artery; ECG, electrocardiogram; LBB, left bundle branch block; RBB, right bundle branch block; PTF, p-wave terminal force.*

**TABLE 3:** Cardiac work-up [*n* (%) or median (IQR)].

	Sinus rhythm	AFDAS	*p*
CEM data (*n* = 224)	199.00 (88.84)	25.00 (11.16)	
PAC, day 1	1.00 (0.00–4.75)	9.00 (2.00–26.50)	< 0.001
NSSVT, day 1	0.00 (0.00–1.00)	3.00 (1.00–9.00)	< 0.001
NSVT, day 1	3.50 (1.00–7.00)	11.00 (2.50–23.00)	< 0.001
pNN50 (*n* = 158)	2.00 (0.00–9.55)	14.11 (4.13–22.39)	< 0.001
Sinus variability (SDNN) (*n* = 158)	29.08 (21.19–46.77)	41.67 (27.13–72.50)	0.002
pNN50 ≥ 11 (*n* = 158)	25.00 (21.40)	25.00 (61.00)	< 0.001
SDNN ≥ 38 (*n* = 158)	36.00 (30.80)	27.00 (65.90)	< 0.001
**Echocardiographic data**			
LA diameter, cm	3.60 (3.20–3.93)	4.10 (3.60–4.40)	< 0.001
LA surface, cm^2^	17.70 (15.05–22.20)	20.80 (17.03–25.87)	0.001
LA volume, mm^3^	45,00 (35.30–61.25)	60.90 (42.00–81.60)	< 0.001
LAVI, ml/m^2^	25.84 (19.45–33.25)	35.80 (25.87–43.86)	< 0.001
LAVI ≥ 33.5 ml/m^2^ (*n* = 188)	32.00 (23.50)	32.00 (61.50)	< 0.001
LVEF,%	60.00 (56.00–66.15)	59.90 (55.00–63.50)	0.030
PFO	26.00 (16.50)	2.00 (3.00)	0.004

*SR, sinus rhythm; AF, atrial fibrillation; PAC, premature atrial contractions; NSSVT, non-sustained supra ventricular tachycardia; NSVT, non-sustained ventricular tachycardia; LAVI, left atrial indexed volume; LVEF, left ventricular ejection fraction; PFO, patent foramen ovale.*

**TABLE 4:** Univariate and multivariate analysis of AFDAS predictors.

	Univariate			Multivariate		
Variable	OR	95% CI	*p*	OR	95% CI	*P*
**Model 1 (*n* = 240)**						
Age ≥ 77 yo	7.45	4.06–13.67	< 0.001			
Female sex	1.96	1.13–3.45	0.016			
Active smoking	0.26	0.10–0.63	0.003			
Insular stroke	2.57	1.37–4.81	0.003			
LAVI ≥ 33.5 ml/m^2^	5.20	2.62–10.32	< 0.001	2.982	1.34–6.63	0.007
PFO	0.16	0.04–0.68	0.013			
NT-pro-BNP ≥ 290 pg/ml	8.39	4.34–16.26	< 0.001	3.950	1.75–8.89	0.001
Galectin-3 ≥ 9 ng/ml	3.49	1.61–7.57	0.002	3.101	1.04–9.25	0.042
ST2 ≥ 18,350 pg/ml	2.59	1.47–4.55	0.001			
OPG ≥ 887 pg/ml	3.44	1.85–6.41	< 0.001	2.338	1.02–5.62	0.046
GDF15 ≥ 1,320 pg/ml	2.56	1.35–4.83	0.004			
**Model 2 (*n* = 158)**						
Age ≥ 77 yo	6.07	2.81–13.11	< 0.001			
LAVI ≥ 33.5 ml/m^2^	4.37	1.88–10.17	0.001			
NT-pro-BNP ≥ 290 pg/ml	6.83	3.05–15.32	< 0.001	4.676	1.66–13.21	0.004
Galectin-3 ≥ 9 ng/ml	7.04	2.04–24.25	0.002	6.587	1.53–28.38	0.011
ST2 ≥ 18,350 pg/ml	2.56	1.23–5.36	0.012			
OPG ≥ 887 pg/ml	2.99	1.34–6.67	0.007	3.350	1.06–10.59	0.040
GDF15 ≥ 1,320 pg/ml	2.72	1.19–6.23	0.018			
PNN50 ≥ 11	5.75	2.67–12.39	< 0.001	8.260	2.80–24.41	< 0.001
SDNN ≥ 38*	4.34	2.04–9.33	< 0.001			

AF, atrial fibrillation; CI, confidence interval; HR, hazard ratio.*Not included in the multivariate analysis due to collinearity with PNN50 (R = -0.74).

Compared with sinus rhythm patients, the patients who developed AFDAS were older (*p* < 0.001), were more often women, were less often active smokers (*p* = 0.001), had a higher NIHSS score on admission (*p* = 0.001), and were likely to have a premorbid mRS ≥ 2 (*p* < 0.001). The CHA_2_DS_2_VASc score at admission (calculated without including the current episode of stroke) was also higher in the AFDAS group (*p* < 0.001), and AFDAS patients were more likely to have undergone acute revascularization therapy by thrombolysis and/or mechanical thrombectomy (*p* = 0.002) (Table 1). On brain CT-scan imaging, the stroke location of patients with AFDAS more frequently involved the superficial middle cerebral artery territory (*p* < 0.001), especially when the insula was involved (*p* = 0.003).

AFDAS patients also had higher blood levels of NT-pro-BNP (*p* < 0.001) (Table 2). Plasma levels of OPG (*p* < 0.001), galectin-3 (*p* = 0.026), GDF 15 (*p* = 0.001) and ST2 (*p* = 0.027) were higher in AFDAS patients. Patients with AFDAS more frequently had LA dilatation as assessed by increased left atrial indexed volume (LAVI) (*p* < 0.001), and had lower LVEF (*p* = 0.030) (Table 3).

In patients without evidence of AF at admission or during CEM in the stroke unit, (*N* = 158), pNN50 (*p* < 0.001) and SDNN (*p* = 0.007), both calculated on the first day of the CEM, were higher in patients who subsequently developed AFDAS. AFDAS was also associated with higher burden of premature atrial contractions (PAC), (*p* < 0.001), non-sustained supraventricular tachycardias (*p* < 0.001) and premature ventricular contractions (PVC) (*p* < 0.001) on CEM.

We performed ROC curves analyses to assess the relationship and the best cut-off values between AF and the biological, imaging and electrocardiographic markers of atrial cardiopathy. After ROC curve, the best predictive value for AF was 887 pg/ml for OPG, 18,350 pg/ml for ST2, 1,320 pg/ml for GDF-15, 11 ng/ml for galectin-3, 290 pg/ml for NT-pro-BNP, 33.5 ml/m^2^ for LAVI, 38 for SDNN and 11 for pNN50.

During the 6 months of follow-up, there were significantly more deaths in the AFDAS group than in the sinus rhythm group [10 (13%) vs. 3 (2%), *p* = 0.001]. There was also a trend toward more frequent bleeding in AF patients at 6 months. There was no difference in the recurrence rate of stroke or TIA.

### Predictive Models for Atrial Fibrillation Detected After Stroke

Two multivariate models were performed, one to predict all recorded AFDAS (model 1) and another model (model 2) focusing on AFDAS diagnosed after patients’ stay in the stroke unit (> 48 h usually), including HRV variables (Table 4).

In model 1, among the variables significantly associated with AF in bivariate analysis, galectin-3 ≥ 9 ng/ml [OR 3.10; 95% CI (1.03–9.254), *p* = 0.042], NT-pro-BNP ≥ 290 pg/ml [OR 3.950; 95% CI (1.754–8.892, *p* = 0.001], OPG ≥ 887 pg/ml [OR 2.338; 95% CI (1.015–5.620), *p* = 0.046] and LAVI ≥ 33.5 ml/m2 [OR 2.982; 95% CI (1.342–6.625), *p* = 0.007] were independently associated with AFDAS.

In model 2, including HRV variables, galectin-3 ≥ 9 ng/ml [OR 6.587; 95% CI (1.529–28.376) *p* = 0.011], NT-Pro-BNP ≥ 290 pg/ml [OR 4.676; 95% CI (1.655–13.210), *p* = 0.004], OPG ≥ 887 pg/ml [OR 3.350; 95% CI (1.060–10.590) *p* = 0.040] and pNN50 ≥ 11 [OR 8.260; 95% CI (2.795–24.406), *p* < 0.001] were independently associated with AFDAS after discharge from the stroke unit.

**FIGURE 2:**
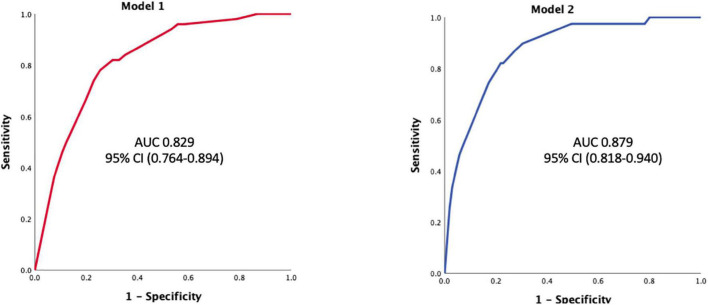
ROC curve for models 1 and 2. Model 1 associates galectin-3 = 9 ng/ml; NT-pro-BNP ≥ 290 pg/ml; OPG ≥ 887 pg/ml and LAVI ≥ 33.5 ml/m^2^ for all AFDAS prediction (*n* = 240). Model 2 associates galectin-3 ≥ 9 ng/ml; NT-Pro-BNP ≥ 290 pg/ml; OPG ≥ 887 pg/ml *and* pNN50 ≥ 11 for AFDAS occurring after a stay in the stroke unit: AUC, area under the curve. OPG, osteoprotegerin.

The ROC curves of these models illustrate their predictive performance for AFDAS in our cohort (Figure 2) [model 1: AUC 0.829, 95% CI (0.764–0.894); model 2: AUC 0.879, 95% CI (0.818–0.940)]. For model 1, the positive predictive value was 63% and the negative predictive value was 80%. For model 2, the positive predictive value was 71% and the negative predictive value was 83%.

## Discussion

**FIGURE 3:**
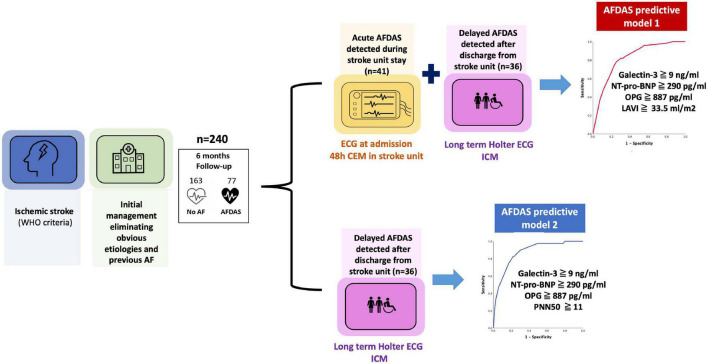
SAFAS study main results.

The main results of this prospective study in ischemic stroke patients without previous AF or an obvious etiology at admission are as follows (Figure 3):

• Our sequential, continuous and early rhythm monitoring approach detected AFDAS in 32% of patients at 6 months of follow-up.
• Several clinical and imaging parameters, novel blood biomarkers (such as galectin-3 and osteoprotegerin), and electrocardiographic parameters such as PNN50 were associated with AFDAS. We can thus confirm the association between atrial cardiopathy markers and the new concept of AFDAS.
• The use of a multimodal approach based on the 3 key determinants of arrhythmia resulted in highly predictive models of AFDAS. These models could help to better stratify the screening strategy for AFDAS, especially for the use of ICM after hospitalization for stroke.

### Left Atrial Substrate: Morphological, Biological and Electrical Assessment

Several studies have demonstrated the association between LA dilatation and AF. Some have even suggested that increased LAVI could be associated with stroke independently of AF onset (14). LA enlargement may promote blood stasis, endothelial damage and thrombus formation (15). This hypothesis is supported by recent data suggesting that atrial fibrosis increases thromboembolic risk regardless of atrial rhythm: this is the concept of atrial cardiopathy (16, 17). In our study, we found a strong association between LA remodeling and AFDAS, as assessed by LA dilatation (increased LA diameter, surface and LAVI). The optimal threshold of LAVI associated with the risk of AF occurrence was 33.5 ml/m^2^. This threshold corresponds to the threshold of mild dilatation on echocardiography (18) and is close to the Carrazco study cut-off (30 ml/m^2^) (19), but lower than the threshold of 44–45 ml/m^2^ found in some studies that have shown this association in the context of cryptogenic stroke (20).

In addition to LA dimensions, several research teams have suggested the use of electrocardiographic markers of atrial cardiopathy such as PTFV_1_. This parameter has been associated with increased risk of AF after adjustment for other markers of atrial cardiopathy such as LA dimensions and NT-pro-BNP (21, 22). PTFV_1_ may be a marker of atrial changes such as fibrosis and elevated filling pressure that are not fully revealed by echocardiographic or serum biomarker assessments. In our study, in a sample of patients (*n* = 111) in whom these measurements were feasible, we did not find a significant association between these ECG markers and AFDAS, contrary to LA dilatation or biomarkers of atrial substrate. This could be related to the population size or to the inclusion of more powerful markers of atrial cardiopathy in the prediction models.

Moreover, three biomarkers of atrial cardiopathy were associated with AFDAS in our study:

- In both predictive models, galectin-3, a biomarker of fibrosis (23), was independently associated with AFDAS. Galectin-3 blood levels are increased in AF patients, are independently correlated with LA volume (24) and predict AF onset and recurrence after AF ablation (25). Although the exact pathophysiological mechanisms by which galectin-3 promotes AF are still unclear, it appears to play an important role in fibrotic processes. It could therefore be a potential marker of interest for atrial cardiopathy.
- Osteoprotegerin is a protein is expressed in endothelial and smooth muscle cells and is involved in the regulation of the inflammatory response and remodeling of the extracellular matrix (26). Its association with AF was only recently suggested. Cao et al. showed that AF patients had higher atrial gene expression of the OPG/RANK/RANKL axis and a higher RANKL/OPG ratio, particularly in paroxysmal AF (27). This expression was also well correlated with markers of atrial remodeling including markers of apoptosis, pro-inflammatory factors, and the matrix metalloproteinase/tissue inhibitors of metalloproteinases system regulating extracellular matrix degradation (28). OPG could therefore be associated with AF through atrial remodeling processes, and could be suggested as a new marker of atrial cardiopathy.
- NT-proBNP levels are increased in stroke patients diagnosed with AF, and are reported to be higher in case of cardioembolic stroke (29, 30). In our study, NT-proBNP values over 290 pg/ml were significantly associated with the occurrence of AFDAS in both models, a threshold comparable to another study on cryptogenic stroke (30). Moreover, NT-proBNP levels > 250 pg/ml were used as a surrogate of atrial cardiopathy in the ARCADIA study (31). Finally, in the TARGET-AF study of stroke patients whose AF was detected by early and prolonged heart rate recordings, Suissa et al. suggested that low BNP levels could virtually exclude the risk of secondary AF (32). These findings suggest that this biomarker could be of great clinical value for targeted AF screening given its strong and independent predictive value of AFDAS in our study.

Taken together, these results suggest that these biomarkers could be of great clinical value for targeted AF screening and atrial cardiopathy diagnosis, given their strong and independent predictive value of AFDAS.

### Modulator

The ANS acts as a modulator of AF onset through the modulation of atrial electro-physiological properties. Adami et al. demonstrated that patients with R-R interval variability after ischemic stroke had an increased risk of AF (33). In our second multivariate model, pNN50 ≧ 11 was associated with an eightfold higher risk of AFDAS in patients without previous evidence of AF on ECG at admission or during CEM in the stroke unit. This analysis is particularly interesting because these data can be automatically extracted from CEM data in the stroke unit, making it feasible in routine clinical practice. We suggest that, if confirmed in further studies, temporal HRV measurements could be included in the cardiac work-up after stroke, similar to LAVI or NT-pro-BNP levels.

### Atrial Fibrillation Triggers

Inflammation plays a role in the initiation, persistence and recurrence of AF. In our study, several inflammatory mediators know (ST2, GDF15, CRP) were associated with the occurrence of AF in bivariate analysis but did not remain significantly associated with AF in our predictive models. This suggests that the pathophysiology of AFDAS is more likely to involve chronic remodeling (atrial cardiopathy) rather that acute triggers such as inflammation or acute myocardial dysfunction.

Finally, the prognostic significance of AFDAS remains uncertain. Further studies are needed to assess the benefit of anticoagulants in AFDAS on the risk of stroke recurrence. In this regard, the ARCADIA trial, which aims to compare an anticoagulant strategy with apixaban vs. aspirin in patients with cerebral infarction of undetermined etiology with recognized markers of atrial cardiopathy (*P*-wave terminal force > 5,000 μV.ms in V1, serum NT-pro-BNP > 250 pg/mL, or left atrial diameter index ≥ 3 cm/m^2^) (31) should add significant knowledge to this clinical issue.

### Limitations

Our study has certain limitations. First, it was a monocentric study on a population based exclusively at the Dijon University Hospital, and we excluded patients referred by other hospitals, which limited the number of inclusions. In addition, some patients with ischemic stroke were not admitted to the stroke unit and therefore could not be included. The study follow-up was limited to 6 months in the study design, in contrast to some studies that completed up to 3 years of monitoring (1, 19). This could have led to an underestimation of AF incidence and to false negatives in the sinus rhythm group. However, in the study by Carrazco et al. 80% of AF cases were diagnosed within the first 6 months of screening (19).

## Conclusion

In order to improve the cost-effectiveness of long-term external Holter recordings and ICM implantations, it is essential to target the patients most at risk of AFDAS, who should benefit from a prolonged rhythm screening strategy. Our multimodal approach combining imaging, electrocardiography and original biological markers of atrial cardiopathy resulted in good predictive models for AFDAS at 6-month follow-up. These results also suggest that AFDAS is probably not be an epiphenomenon related to the acute stroke but rather related to underlying atrial cardiopathy. Further studies are needed to evaluate the embolic risk and the indication for anticoagulation in these AFDAS patients.

## Data Availability Statement

The raw data supporting the conclusions of this article will be made available by the authors, without undue reservation.

## Ethics Statement

The studies involving human participants were reviewed and approved by the CPP Sud Méditerranée I n°2018-A00345-50. Written informed consent for participation was not required for this study in accordance with the national legislation and the institutional requirements.

## Author Contributions

LG, GDu, AS, AM, GDo, RD, MG, YB, CV, and CG: substantial contributions to the conception, design of the work, the acquisition, analysis, and interpretation of data for the work. KB, TP, CV, YB, and CG: drafting the work and revising it critically for important intellectual content. All authors have substantially approved its submission to the journal and are prepared to take public responsibility for the work.

## Conflict of Interest

The authors declare that the research was conducted in the absence of any commercial or financial relationships that could be construed as a potential conflict of interest.

## Publisher’s Note

All claims expressed in this article are solely those of the authors and do not necessarily represent those of their affiliated organizations, or those of the publisher, the editors and the reviewers. Any product that may be evaluated in this article, or claim that may be made by its manufacturer, is not guaranteed or endorsed by the publisher.
